# Oral Health‐Related Quality of Life in Dutch Adults With Osteogenesis Imperfecta

**DOI:** 10.1111/odi.15163

**Published:** 2024-10-22

**Authors:** Emmanuelle de Kuijper‐Timmermans, Lieke Blokland, Maurits de Kuijper, Stanimira Kalaykova, M. Carola Zillikens, Natasha M. Appelman‐Dijkstra, Koert Gooijer, Arjan Harsevoort, Guus J. M. Janus

**Affiliations:** ^1^ Vogellanden Center of Rehabilitation Medicine & Special Care in Dentistry Zwolle The Netherlands; ^2^ Department of Oral and Maxillofacial Surgery University of Groningen, University Medical Center Groningen Groningen The Netherlands; ^3^ Radboud University Medical Center Nijmegen The Netherlands; ^4^ Department of Restorative Dentistry, Center for Dentistry and Oral Hygiene University Medical Center Groningen, The University of Groningen Groningen The Netherlands; ^5^ Department of Dentistry Radboud University Medical Center Nijmegen The Netherlands; ^6^ Erasmus MC Bone Center, Department of Internal Medicine Erasmus University Medical Center Rotterdam The Netherlands; ^7^ Division Endocrinology and Center for Bone Quality, Department of Internal Medicine Leiden University Medical Center Leiden The Netherlands; ^8^ Expert Center for Adults With Osteogenesis Imperfecta Isala Hospital Zwolle The Netherlands

**Keywords:** Dentinogenesis Imperfecta, Oral Health Impact Profile, Oral health‐related quality of life, Osteogenesis Imperfecta, rare bone condition, rare disease, self‐reported oral health

## Abstract

**Objective:**

To explore the oral health‐related quality of life and its possible risk factors among adults with Osteogenesis Imperfecta using the Oral Health Impact Profile (OHIP)‐49 questionnaire. Secondary objectives were to investigate the impact of self‐reported Osteogenesis Imperfecta, Dentinogenesis Imperfecta, and age on various dental parameters.

**Materials and Methods:**

A cross‐sectional questionnaire was distributed online to 417 Dutch adults with Osteogenesis Imperfecta at three national referral centers. Multivariate linear regression was performed to identify indicators for OHIP‐49 Scores. The effect of Osteogenesis Imperfecta, Dentinogenesis Imperfecta, and age on various dental parameters was investigated using logistic regressions (*p* < 0.05).

**Results:**

One hundred and fifty‐five questionnaires (37.2%) were suitable for analysis. Osteogenesis Imperfecta type III was significantly associated with higher OHIP‐49 scores as compared to type I. Symptoms of temporomandibular disorders, missing teeth or dentures also increased the OHIP‐49 scores significantly. Osteogenesis Imperfecta type IV and increasing age were associated with missing teeth. There was a 31.94 times (95% CI: 8.56–119.13) higher odds of tooth fracture with self‐reported Dentinogenesis Imperfecta.

**Conclusions:**

Adults with Osteogenesis Imperfecta type III report a lower oral health‐related quality of life compared to Osteogenesis Imperfecta type I adults.

## 1 Introduction

Osteogenesis Imperfecta (OI) is a rare connective tissue disorder, also known as brittle bone disease. It is with variable severity characterized by fragile bones, easy susceptible to fracture. This is due to defects in collagen type I biosynthesis (Van Dijk et al. 2010). It is mostly caused by genetic variants in COL1A1 and COL1A2 (Forlino and Marini 2016).

The most frequently employed classification is the Sillence classification, which categorizes OI into four types according to clinical and genetic findings (Sillence, Senn, and Danks 1979): OI type I (mild or no bone deformity), type II (Lethal perinatal OI), type III (progressively severe deforming OI), and type IV (moderate bone deformity). The presentation of OI within these types exhibits significant variability (Byers, Wallis, and Willing 1991). Alternative classifications based on genetic findings have been suggested (Van Dijk et al. 2010; Van Dijk and Sillence 2014). The prevalence of OI is approximately 6–7/100,000 (Steiner and Basel, 2005). The prevalence of OI in the Netherlands was estimated around 850 patients (Storoni et al. 2022).

OI is often associated with oral health‐related symptoms. The most common one is Dentinogenesis Imperfecta (DGI). When DGI is linked to OI, it is categorized as DGI type 1. In teeth affected by DGI, a typical feature is a gray‐blue to brown discoloration, and the otherwise relatively intact enamel is more susceptible to fractures and chipping due to dentin dysplasia. Radiographic indicators of DGI include morphological abnormalities such as bulbous crowns with cervical constrictions, pulpal obliteration, and shortened root radices (Andersson et al. 2017; Thuesen et al. 2018).

In OI the prevalence of DGI is estimated between 8% and 100%, depending on the type of OI. Its prevalence is higher in OI types III and IV (Saeves et al. 2009; Hennekam, Allanson, and Krantz 2010; Thuesen et al. 2018; Hald et al. 2018; Andersson et al. 2017). Other oral health‐related symptoms include dental and skeletal class III malocclusions associated with anterior and lateral open bites, impacted teeth and tooth agenesis (Rizkallah et al. 2013; Shields, Bixler, and El‐Kafrawy 1973; O'Connell and Marini 1999; Saeves et al. 2009; Waltimo‐Sirén et al. 2005).

Based on the experience of our team in providing dental care to this patient group in a specialized setting, many patients report that diagnosis and treatment of oral health‐related problems receive little priority and that oral health professionals may appear reluctant to act on them.

However, due to the specific OI‐related orofacial problems, there is a need for treatment. Patient's complaints that are mentioned during the treatment within our team focus on both the dental esthetics and oral function including frequent tooth fractures and feelings of embarrassment due to issues with teeth or the mouth.

Existing studies on oral health in OI primarily focus on oral health‐related quality of life in children and adolescents (Najirad et al. 2018; Cachia Mintoff, Riddington, and Parekh 2022). These studies have shown an influence of oral manifestations, like DGI, in children with OI on the oral health‐related quality of life. Adults with moderate to severe OI exhibit a slightly greater impact on Oral Health‐Related Quality of Life (OHRQoL) compared to individuals with mild OI (Gjørup et al. 2021).

However, there is still insufficient knowledge regarding the perception of oral health and perceived orofacial esthetics among individuals with OI. Oral health problems in individuals with OI could influence their quality of life and there is a scarcity of studies specifically addressing the impact of oral health on the quality of life of OI.

The Oral Health Impact Profile (OHIP) is a validated scaled index measuring the social consequences of oral disorders, utilizing a theoretical hierarchy of oral health outcomes (Slade and Spencer 1994). Comprising 49 questions, it explores the adverse effects of oral conditions on everyday functioning. This profile is a reliable and valid tool for in‐depth assessment of the social impact of oral disorders, offering potential advantages in both clinical decision‐making and research (Slade 1998; Jones et al. 2004). For the perceived orofacial esthetics, the Orofacial Esthetics Scale (OES) has proved to be a valid instrument (John et al. 2012).

This article is the first part of a set of two publications. The aim of this study was to explore the oral health‐related quality of life and possible risk factors influencing this oral health‐related quality of life among adults with various OI types. Secondary objective was to look into the effect of self‐reported OI type, presence of DGI and age on various dental‐ and health‐ related parameters, such as dental status, fracture of teeth, bisphosphonate use, and the location where dental care was provided. Lastly, the impact of OI type and DGI on the orofacial esthetic scale was investigated.

## 2 Material and Methods

### 2.1 Design

This study was part of a larger study considering self‐reported oral health in adults with OI in the Netherlands. The cross‐sectional study adheres to the guidelines outlined in the Strengthening the Reporting of Observational Studies in Epidemiology (STROBE) for observational studies and was approved by the medical ethical committee of the Isala Hospital (application number 20220806). All participants provided either written or digital informed consent.

### 2.2 Study Population

An online questionnaire was distributed to a total of 417 Dutch adults with OI from February to May 2023. Email invitations were sent to all adults with OI with a known email address who were under the care of three Expert Centers for adults with Osteogenesis Imperfecta in the Netherlands (Isala Hospital, Erasmus Medical Center Rotterdam and Leiden University Medical Center). Furthermore, adults with OI were informed about the research during a meeting organized by the patient federation for OI (Vereniging Osteogenesis Imperfecta, VOI). Only adults that reported having a diagnosis of OI were included. Adults with an inconclusive clinical or genetic diagnosis of OI were excluded.

### 2.3 Questionnaire

A digital questionnaire was constructed in an online electronic data capturing software (ResearchManager). The questionnaire consisted of 110 questions on the domains general information, Oral Health‐Related Quality of Life (OHRQoL), oral esthetics, function and dysfunction, and symptoms that could indicate risk for sleep apnea. The latter is the topic of a second manuscript (Blokland et al. 2024).

General variables consisted of 11 self‐reported items regarding age, gender (M/F/Other), type of OI (I, III, IV, and unknown/other type), the use of bisphosphonates (yes, no, and quitted. When yes: IV/oral and how many years), location of dental care (no care, general dentist, and center for special dental care), perception of the received professional dental care (good, moderate, bad, and not applicable), self‐reported presence of DGI (yes, no, and unknown), fracturing of teeth (yes, no; when yes: more often than once per year), dental situation (intact dentition, partially dentate, presence of implants, denture), perception of the dental/oral situation (good, moderate, and bad) and presence of sleep apnea (yes, no. If yes: treatment yes or no). For the other questions, the questionnaires OHIP‐NL49, OES, and 3Q/TMD were used.

The OHIP‐NL49 is the Dutch validated adaptation of the OHIP‐49, comprising 49 items across seven domains: functional limitations, physical pain, psychological discomfort, physical disability, psychological disability, social disability, and handicap (van der Meulen et al. 2008). Respondents were asked about the frequency of experiencing specific issues related to their teeth, mouth, or dentures for each item. Responses to the 49 questions are rated on 5‐point ordinal scales, ranging from never (0), hardly ever (1), occasionally (2), fairly often (3), to very often (4). The total score ranges from 0 to 196, and domain scores can also be calculated. Higher scores indicate a more compromised oral health‐related quality of life.

The OES‐NL, the Dutch adaptation of the OES, is an eight‐item questionnaire designed to assess self‐perceived orofacial esthetics (Wetselaar et al. 2015). Respondents could express their feelings about the appearance of their face, mouth, teeth, and tooth replacements. The questionnaire addressed seven components (face, tooth shape, tooth color, and gum). Participants provided responses on a numeric rating scale from 0 to 10, ranging from very dissatisfied (0) to very satisfied (10). The total score spans from 0 to 70. The eighth item's score (patient's global assessment) is evaluated separately and ranges from very dissatisfied (0) to very satisfied (10). Higher scores indicate greater patient satisfaction, while lower scores suggest more compromised self‐perceived orofacial esthetics.

The 3Q/TMD consists of three questions on TMD symptoms, to screen for temporomandibular disorders and the need for further assessment (Lövgren et al. 2016). Respondents were categorized as either 3Q‐positive or 3Q‐negative, according to ≥ 1 positive answers or 0 positive answers, respectively.

### 2.4 Statistical Analysis

Data were analyzed using SPSS (IBM statistics, version 29.0). Descriptive statistics of the original dataset were used to characterize the respondents based on gender, self‐reported type of Osteogenesis Imperfecta, location where dental care is provided, dental status, perceived oral health, and bisphosphonate use. Cases with more than 55% missing values were excluded. Multiple imputation was used to address the missing values in the dataset (full conditional model with 50‐fold imputations), after checking for the missing at random assumption (Little's MCAR test). Imputations for the following variables were used: OHIP‐49 (2 respondents had partially missing values), dental status (7.1% missing), TMD‐pain (6.5% missing), sleep apnea (5.2% missing), bisphosphonate use (5.2% missing), location of dental care provided (4.5%), 3Q‐TMD (3.9% missing), gender (3.2% missing), MFIQ (2.6% missing), and age (3.2% missing). A complete OHIP‐49 score was calculated using the sum of all imputed OHIP‐scores. The OHIP‐score was further divided in seven domains (functional limitations, physical pain, psychological discomfort, physical disability, psychological disability, social disability, and handicap). Mean OHIP‐49 scores of a reference group of 663 Dutch dentate patients were used as a reference and plotted together with the mean OHIP‐49 scores of the dentate respondents from this survey. Welch's t tests were conducted with Bonferroni correction to investigate a significant difference between the OHIP‐scores of the dentate reference and dentate OI population. Univariate linear regression with the imputed dataset was performed to identify potential indicators for the complete OHIP‐49 score. Indicator variables with a *p* < 0.05 were incorporated in a multivariate linear regression model, after checking the assumptions. Categorical variables were dummy‐coded. The assumption of heteroscedasticity was violated, and the OHIP‐49 scores were transformed into inverse scores to meet this assumption. Since the transformed OHIP‐49 scores still resulted in the same significant indicators, for the ease of interpretation, the multivariate linear regression of the untransformed imputed OHIP‐49 scores were presented. Logistic regressions were performed to analyze the effect of self‐reported Osteogenesis Imperfecta type, self‐reported presence of DGI, age and, when appropriate, complete OHIP scores on dental status (dentate or missing teeth/denture), self‐reported frequently fracturing of teeth (yes or no), bisphosphonate use (yes or no), the location of where dental care was provided (inside or outside hospital), and quality of the dental care received (good or poor/bad). Multicollinearity and linearity in the logit of the variable age and complete OHIP scores were checked. For the 3Q‐TMD, a dichotomous variable was constructed with score 0 representing no TMD symptoms and scores 1‐2‐3 an increased risk of TMD symptoms. For the Oral Esthetic scale, a summary score was computed. After checking assumptions, multivariate linear regression was done with OI type, presence of DI, dental status, and age as dependent variables. An alpha of 0.05 was considered significant in all aforementioned tests.

## 3 Results

### 3.1 Respondent's Characteristics

Of the 417 adults who received the questionnaire, two respondents were excluded because they did not (self‐)report to have the diagnosis of OI (but osteoporosis). A total of 155 questionnaires (37.2%) were suitable for analysis. Table 1 describes the characteristics of the respondents. Mean age was 47.1 years (SD ± 14.6 years; minimum 20 years; maximum 76 years). When respondents reported to use bisphosphonates, the median time of use was 3.0 years (interquartile range: 5 years; minimum 0 years (recently started); maximum 20 years).

**TABLE 1:** Characteristics of the respondents that completed the OHIP‐49 questionnaire (percentages are given as a relative percentage of the total respondents).

Variables	Category	*n* (%)
Gender	Male	57 (36.8)
	Female	93 (60.0)
	Missing	5 (3.2)
OI/DGI	*Type I*	84 (54.2)
	Type 1 with DGI	8 (5.2)
	Type 1 without DGI	41 (26.5)
	Type 1 unknown DGI	35 (22.6)
	*Type III*	18 (11.6)
	Type III with DGI	6 (3.9)
	Type III without DGI	5 (3.2)
	Type III unknown DGI	7 (4.5)
	*Type IV*	24 (15.5)
	Type IV with DGI	12 (7.7)
	Type IV without DGI	7 (4.5)
	Type IV unknown DGI	5 (3.2)
	*Other*	29 (18.7)
	Other with DGI	6 (3.9)
	Other without DGI	7 (4.5)
	Other unknown DGI	16 (10.3)
Dental status	*Intact dentition*	47 (30.3)
	OI Type I	35 (22.6)
	OI Type III	8 (5.1)
	OI Type IV	2 (1.3)
	OI Other	2 (1.3)
	*One or more missing teeth*	89 (57.4)
	OI Type I	42 (27.0)
	OI Type III	10 (6.5)
	OI Type IV	19 (12.3)
	OI Other	18 (11.6)
	*Denture*	8 (5.2)
	OI Type I	5 (3.2)
	OI Type III	0 (0.0)
	OI Type IV	2 (1.3)
	OI Other	1 (0.7)
	*Missing*	11 (7.1)
Dental implants	*Yes*	20 (12.9)
	OI Type I	9 (5.8)
	OI Type III	1 (0.7)
	OI Type IV	4 (2.5)
	OI Other	6 (3.9)
	*No*	135 (87.1)
Dental care facility	*General practice*	103 (66.5)
	OI Type I	65 (41.9)
	OI Type III	9 (5.8)
	OI Type IV	13 (8.5)
	OI Other	16 (10.3)
	*Center for special dental care*	29 (18.7)
	OI Type I	9 (5.8)
	OI Type III	8 (5.2)
	OI Type IV	8 (5.2)
	OI Other	4 (2.5)
	*None*	16 (10.3)
	OI Type I	10 (6.5)
	OI Type III	1 (0.7)
	OI Type IV	3 (1.8)
	OI Other	2 (1.3)
	*Missing*	7 (4.5)
Bisphosphonate use	Intravenously	9 (5.8)
	Orally	26 (16.8)
	Discontinued	40 (25.8)
	None	72 (46.4)
	Missing	8 (5.2)
View on quality of dental care provided by the dentist	Good	106 (68.4)
	Poor/Bad	31 (20.0)
	Not applicable	11 (7.1)
	Missing	7 (4.5)
Symptoms of TMD	No	98 (63.2)
	Symptoms of TMD (≥ 1 positive question(s))	48 (31.0)
	Missing	9 (5.8)
Frequent fracture of teeth	*No DGI*	
	Yes	9 (5.8)
	No	51 (32.9)
	Missing	95 (61.3)
	*DGI*	
	Yes	27 (17.4)
	No	5 (3.2)
	Missing	123 (79.4)

Abbreviations: DGI = Dentinogenesis Imperfecta, OI = Osteogenesis Imperfecta.

### 3.2 OHIP‐49

**FIGURE 1:**
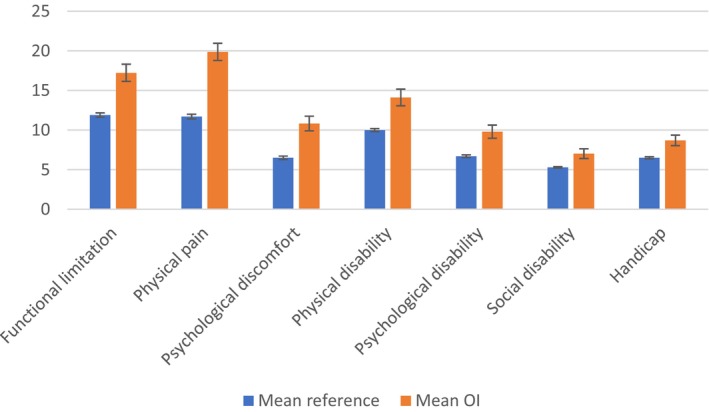
Mean OHIP‐49 scores of the pooled 50‐fold imputations (with 95% CI) per domain for dentate Osteogenesis Imperfecta adults (*n* = 147; orange bars; mean age: 45.6 ± 14.4 years) and a reference group of 663 Dutch dentate adults without Osteogenesis Imperfecta (mean age: 50.6 ± 12.6 years) (Kieffer, Verrips, and Hoogstraten 2011; blue bars).

Overall results of the OHIP‐49 questionnaire are listed in Table 2. After univariate analyses, variables OI type, DGI, dental status, and presence of TMD symptoms were included in the multivariate model. Multivariate linear regression showed a significant increase in OHIP‐49 scores for OI type III adults as compared to OI type I, when a respondent reported missing teeth or signs of TMD symptoms according to the 3Q/TMD (see Table 3). In Figure 1, mean OHIP‐49 scores per domain are plotted for dentate OI adults and a reference group of 663 Dutch dentate adults without OI (Kieffer, Verrips, and Hoogstraten 2011). Dentate OI patients had significantly higher scores on all OHIP‐domains compared to a reference population with large effect sizes (*d* > 0.90).

**TABLE 2:** Descriptive statistics for all respondents (*n* = 155) per OHIP‐49 category.

OHIP	Mean (SD)	Minimum‐maximum	95% CI Lower bound	95% CI Upper bound
Complete OHIP	88.30	29.0–212.0	82.99	93.61
Functional limitation	17.47	8.0–38.0	16.43	18.51
Physical pain	20.01	10.0–41.0	18.97	21.06
Psychological discomfort	10.91	0.0–25.0	10.03	11.80
Physical disability	14.18	0.0–39.0	13.19	15.17
Psychological disability	9.88	0.0–30.0	9.08	10.68
Social disability	7.06	0.0–25.0	6.50	7.66
Handicap	8.76	0.0–26.0	8.11	9.41

**TABLE 3:** Results for the multivariate linear regression model of the imputed dataset.

Variables	B	95% CI Lower bound	95% CI Upper bound	*p*‐value
Constant	56.57	46.30	66.81	0.00
OI type III (ref. type I)	**16.21**	**1.11**	**31.30**	**0.03**
OI type IV (ref. type I)	−3.33	−17.34	10.68	0.64
OI type other (ref. type I)	10.54	−2.06	23.14	0.10
DGI (ref. no DGI)	10.62	−2.73	23.98	0.12
Unknown DGI (ref. no DGI)	9.68	−0.66	20.01	0.07
TMD present (ref. no TMD)	**23.14**	**13.36**	**32.91**	**0.00**
Dentate, missing teeth (ref. intact dentition)	**20.16**	**9.32**	**31.01**	**0.00**
Full denture without implants (ref. intact dentition)	**36.52**	**14.19**	**58.84**	**0.00**

*Note:* Bold variables significantly impact the OHIP‐49 score.

Abbreviations: *B* = beta, ref. = reference category.

### 3.3 Dental Status

Respondents reporting type IV OI had a higher odds of missing teeth or having dentures as compared to type I respondents (OR: 12.05; 95% CI: 1.88–77.41; *p* = 0.01). Also, an increase in 1 year of age had a higher odds of missing teeth or having a denture (OR: 1.11; 95% CI: 1.06–1.17; *p* = 0.00).

### 3.4 Fracture of Teeth

Self‐reported type of OI did not influence the reported fracture of teeth. When a respondent reported DGI, there was a 31.94 times odds for tooth fracture as compared to no DGI (95% CI: 8.56–119.13; *p* = 0.00). This was also the case when the presence of DGI was unknown (OR: 3.85; 95% CI: 1.42–10.42; *p* = 0.01). Every 1‐year increase in age also slightly increased the odds for tooth fracture (OR: 1.04; 95% CI: 1.00–1.08; *p* = 0.03).

### 3.5 Bisphosphonate Use

Compared to self‐reported OI type I respondents, type III respondents had a higher odds of prior or current bisphosphonate use (OR: 10.19; 95% CI: 2.14–48.59; *p* = 0.00). Increase in age and the presence of DGI had no significant effect (OR: 1.01; 95% CI: 0.99–1.04; *p* = 0.41 and OR: 0.96; 95%CI: 0.44–2.06; *p* = 0.91).

### 3.6 Location and Quality of Dental Care

Respondents reporting DGI had a higher odds of receiving dental care inside a hospital setting as compared to no DGI (OR: 32.31; 95% CI: 7.86–132.88; *p* < 0.00). OI type III was also associated with a higher odds as compared to type I (OR: 4.57; 95% CI: 1.02–20.54; *p* = 0.047). Age had no significant effect (OR: 0.99; 95% CI: 0.95–1.03; *p* = 0.54). The rated quality of received dental care was not significantly different inside or outside the hospital setting, although there was a trend that care outside a hospital setting had a higher odds of less perceived quality of care (OR: 8.74; 95% CI: 0.90–84.62; *p* = 0.06). An increase in OHIP‐score was slightly associated with less perceived quality of care (OR: 1.03; 95% CI: 1.01–1.04; *p* = 0.00). Age did not influence the perceived quality of care (OR: 0.99; 95% CI: 0.96–1.03; *p* = 0.76).

### 3.7 Oral Esthetic Scale (OES)

The mean for the total OES‐score was 53.63 points (± 15.34 points). In Table 4, the scores per OES‐item are summarized. Multivariate linear regression showed a significant negative effect of self‐reported OI type III. On average, the respondents scored 11.34 points less on the OES‐scale as compared to the respondents reporting OI type I (*p* = 0.01). The presence of DGI, an increase in age, or missing teeth did not affect the OES score, respectively *B* = 0.49, 95% CI: −6.41 to 7.40 (*p* = 0.89), *B* = 0.13, 95% CI: −0.06 to 0.33 (*p* = 0.17) and *B* = −0.73, 95% CI: −7.03 to 5.76 (*p* = 0.82).

**TABLE 4:** Descriptive statistics for all respondents (*n* = 155) for the Oral Esthetic Scale (OES).

OES‐item	Mean (SD)	Minimum–maximum	95% CI Lower bound	95% CI Upper bound
Facial appearance	7.23 (1.86)	0–10	6.94	7.53
Appearance of profile	7.13 (1.97)	0–10	6.82	7.44
Mouth's appearance	6.58 (2.41)	0–10	6.20	6.96
Rows of teeth appearance	6.35 (2.53)	0–10	5.95	6,75
Shape/form teeth	6.56 (2.26)	0–10	6.21	6.92
Color teeth	5.88 (2.60)	0–10	5.47	6.29
Gum's appearance	7.14 (1.97)	0–10	6.83	7.45
Overall appearance	6.75 (1.98)	0–10	6.44	7.06

## 4 Discussion

There was a significant increase in OHIP‐49 scores among OI type III adults compared to those with OI type I indicating a lower Oral Health‐Related Quality of Life (OHRQoL). Signs of TMD symptoms and missing teeth also increased the OHIP‐49 scores significantly. Presence of DGI resulted in a 31.94 times higher odds of tooth fracture compared to respondents not reporting DGI. Adults with OI type III reported a significantly lower OES score compared to those with OI type I. Furthermore, adults reporting OI type IV had higher odds of missing teeth or having a denture compared to OI type I adults.

In comparison to 663 healthy individuals from the Dutch general population (Kieffer, Verrips, and Hoogstraten 2011), the mean OHIP score is higher (poorer) across all seven domains for the dentate OI respondents. The mean scores in the current study differed by 1.66 points in the social disability domain to an increase of 8.61 points increase in the domain of physical pain. While a direct one‐to‐one comparison is not feasible, these findings suggest a diminished Oral Health‐Related Quality of Life for Dutch dentate adults with Osteogenesis Imperfecta compared to the general Dutch dentate population.

Consistent with a Danish study on OI adults (Gjørup et al. 2021), the current study reveals the highest OHIP scores in the domains of functional limitation and pain, followed by domains related to psychological discomfort and physical disability. The lowest scores are observed in the domains of psychological disability, social disability, and handicap.

A poorer OHRQoL was observed among adults with OI type III in comparison to those with OI type I. Similarly, a study focusing on OHRQoL in children and adolescents in North America (Najirad et al. 2018) also noted that adolescents with OI type III exhibited a more negative overall OHRQoL profile as compared to OI type I, particularly in the domain of functional limitation. However, a separate UK‐based study in adolescents (Cachia Mintoff, Riddington, and Parekh 2022) did not reveal significant differences between mild and severe types of OI.

The observation of a lower OHRQoL in adults with OI type III compared to those with OI type I may be attributed to several factors. Firstly, this could be linked to a more significant growth impairment and altered craniofacial shape individuals with OI type III due to distinct growth deficiencies and more prevalent class III malocclusions (Waltimo‐Sirén et al. 2005). Moreover, DGI and the retention of second molars are more frequently observed in adults with OI type III compared to type I (Andersson et al. 2017). Lastly, OI type III adults exhibited the highest prevalence of missing or unerupted teeth (Taqi et al. 2021). The previously mentioned factors could potentially lead to functional consequences such as chewing problems, fractures, and esthetic concerns.

DGI in adults is primarily associated with OI type III and IV (Saeves et al. 2009; Patel et al. 2015). This pattern was also observed in the current study, where 9.5% of type I respondents reported DGI, while 33.3% and 50% of adults with type III and IV, respectively, indicated having DGI. Despite the results not reaching significance, a trend was observed of a decline in OHRQoL among individuals with DGI as compared to individuals without DGI. DGI can impact OHIP scores in at least five domains: functional limitation, physical pain, psychological discomfort, physical disability, and psychological disability. This is because of complications such as altered appearance, chewing difficulties due to tooth fractures or the fear of tooth fractures. The increased risk of tooth fractures may also result in discomfort while eating or avoiding certain foods. The distinct gray‐blue to brown appearance of teeth affected by DGI may lead individuals to refrain from smiling and experiencing embarrassment about their appearance. It is noteworthy that the presence of DGI did not influence the OES score in the current study.

Regarding the higher odds for tooth fracture as compared to respondents without DGI, a study conducted by Ibrahim et al. (2019) identified abnormalities within the dentin, such as reduced collagen density, a decrease in fiber quantity, and irregular shapes of collagen fibers. These abnormalities may play a role in the observed decrease in dentin hardness and the diminished modulus of elasticity. The diminished elasticity of peritubular collagen fibers and the quality of the collagen fibrils are considered preliminary indicators for understanding the structural defects (Gadi, Chau, and Parekh 2023). Consequently, these defects contribute to the reduced structural and mechanical properties of dentin in teeth affected by DGI in association with OI.

However, as far as we know, there is currently no research available regarding the frequency of tooth fractures, dental wear, and the longevity of restorations in cases of DGI. Not to mention evidence‐based restorative strategies and recommendations to restore these teeth.

There was a notable increase in OHIP‐49 scores when a patient reported missing teeth or when a patient had a full denture without implants. An elevated OHIP‐49 score was also seen in healthy adults with full dentures (Awad et al. 2000). Gjørup (Gjørup et al. 2021) discovered a negative association between the number of teeth in the oral cavity and some OHIP domain scores, indicating that the location of absent teeth and the type of prosthesis, if present, may impact OHRQoL.

Additionally, there was a significant increase in OHIP‐49 scores when an individual exhibited symptoms of TMD. In the general population, experiencing signs of TMD has been associated with a significant impact of OHRQoL (Qamar et al. 2023). This impact on OHRQoL may originate from the pain, discomfort, difficulty in eating, speaking and other orofacial functions, and overall impairment in carrying out daily activities that adults with TMD may experience.

Most respondents rated the quality of dental care as good (68.4%), while 20% were dissatisfied, perceiving it as poor or bad. Increased OHIP scores were significantly linked to lower perceived quality of care, indicating a decline in OHRQoL. Although there is limited research on this topic within the general population, a study on dentist–patient relationships in Australian adults found that higher satisfaction and lower dental fear were associated with improved OHRQoL (Song et al. 2020). These findings, along with our study, highlight the importance of a good dentist–patient relationship, particularly in the quality of care domain.

Our study's strength lies in addressing a gap in the existing literature by investigating the OHRQoL in adults with OI. This study is among the first to compare the OHRQoL between adults with different types of OI. We extended invitations to a substantial number of Dutch individuals with OI to participate. The response rate for this cross‐sectional questionnaire study was 37.2%, which might be considered fair. However, it is essential to acknowledge the limitations of our study. It is important to note that the outcomes of this study rely on self‐reported information using an online questionnaire. This required patients to be digitally proficient. However, given the broad age range (20–76 years), the risk of selection bias was deemed minimal. Additionally, there is an extreme phenotypic variation within this population, resulting in considerable differences in the severity of OI across various types and a relatively large number of respondents (18.7%) expressing uncertainty regarding their classification into a specific type (referred to as ‘Other’). Ideally a more detailed examination, such as determining whether missing teeth were replaced by implants, fixed dental prostheses or a partial denture, would be more accurate. However, these groups were too small for statistical analysis. For example, only eight out of the total 155 individuals were having full dentures. Therefore, we did not explore these aspects.

Further research is necessary to comprehensively assess oral health in Dutch individuals with OI, potentially through a case–control study. Integrating medical files with the questionnaire responses could enhance the accuracy of the outcomes. A combination of self‐reported data with clinical and radiographic investigations would be desirable, providing a more comprehensive understanding of the potential presence of DGI and dental status. A recent systematic review on health‐related quality of life (HR‐QOL) in adults with OI found that while mental health scores were generally preserved, certain mental health domains showed reductions (Mc Donald et al. 2023). Additionally, physical HR‐QOL, including pain and fatigue, was significantly impacted when compared to a control population. This could also impact the OHRQoL and TMD symptoms. Given the rarity of OI, a multicenter trial that aggregates data from multiple expert centers across different countries would enhance the robustness and generalizability of the findings from this study. In conclusion, this study investigated the Oral Health‐Related Quality of Life among adults with OI. Adults with OI type III exhibit a lower OHRQoL compared to those with OI type I. Additionally, a diminished OHRQoL is identified when adults report signs of TMD symptoms or missing teeth, in contrast to those without these conditions. Additionally, they should be aware that dentate individuals with OI might experience impaired OHRQoL as compared to healthy dentate individuals. The presence of DGI is associated with a higher likelihood of tooth fracture. Furthermore, adults with OI type III experience more compromised self‐perceived orofacial esthetics compared to those with OI type I. The study underscores a significant association between an impaired oral health‐related quality of life and a lower perceived quality of care. Our results highlight the importance of dental care providers paying close attention to oral health and delivering high‐quality care, as it is clear that oral health significantly affects the quality of life for individuals with OI. Healthcare professionals need to recognize the impact of the type of OI, the presence of TMD symptoms, and dental status on the OHRQoL.

## Author Contributions

**Emmanuelle de Kuijper‐Timmermans:** conceptualization, investigation, writing – original draft, methodology, validation, visualization, writing – review and editing, software, project administration, data curation, resources, formal analysis. **Lieke Blokland:** conceptualization, investigation, methodology, validation, visualization, writing – review and editing, software, data curation, resources, project administration. **Maurits de Kuijper:** formal analysis, writing – review and editing, data curation, conceptualization, methodology, writing – original draft. **Stanimira Kalaykova:** methodology, writing – review and editing, formal analysis, conceptualization. **M. Carola Zillikens:** methodology, writing – review and editing, supervision, resources. **Natasha M. Appelman‐Dijkstra:** methodology, resources, supervision, writing – review and editing. **Koert Gooijer:** methodology, writing – review and editing, software, resources, investigation, data curation. **Arjan Harsevoort:** methodology, investigation, software, resources, writing – review and editing, data curation, conceptualization. **Guus J. M. Janus:** resources, writing – review and editing, supervision, investigation, methodology, conceptualization.

## Conflicts of Interest

The authors declare no conflicts of interest.

## Data Availability

The data that support the findings of this study are available from the corresponding author upon reasonable request.
